# Occupational stress and mental health of cardiac and noncardiac patients

**DOI:** 10.4103/0972-6748.62270

**Published:** 2009

**Authors:** S. Subramanian, D. V. Nithyanandan

**Affiliations:** Department of Psychology, Bharathiar University, Coimbatore, Tamil Nadu, India

**Keywords:** Heart diseases, Mental health, Occupational stress

## Abstract

**Background:**

Much of the research studies have shown that occupational stress is one of the strong determinant factors of coronary heart diseases among people in general. However, exploring the extent to which the type or nature of ailments and its subsequent risk factors have an effect on the onset of mental health will help evolve suitable preventive measures. The present study attempts to explore the status of mental health and occupational stress with respect to 2 categories of patients: Those who are suffering from cardiac problems and those suffering from noncardiac health problems.

**Materials and Methods:**

Occupational Stress Questionnaire and Mental Health Questionnaire were administered to both cardiac and noncardiac patients. The cardiac group consisted of 40 patients who were being treated at the cardiology department of a reputed hospital, and noncardiac group (40 patients) consisted of outpatients of the same hospital being treated for noncardiac problems like knee pain, headache, etc. Responses to these self-reported questionnaires were subjected to statistical analysis to find out the difference between cardiac and noncardiac groups.

**Results:**

The results revealed that cardiac patients tend to have lower levels of mental health than noncardiac patients. Similarly, cardiac patients were reported to have higher levels of stress due to role ambiguity, powerlessness, intrinsic impoverishment and unprofitability.

**Conclusions:**

The implications of the study were implementation of interventions to improve the internal strength of cardiac patients to overcome various aspects of occupational stress.

Modern age is said to be the age of stress, which affects the global functioning of the individual human being, especially due to the demands of his working environment. Hence the present study tried to understand stress and mental health in relation to coronary heart disease.

Stress can generally be defined as the reaction of individuals to demands (stressors) imposed upon them that refer to situations where the well-being of individuals is detrimentally affected by their failure to cope with the demands of their environment (Erkutlu and Chafra, 2006). The term *stress* has many dimensions according to the situation that imposes various demands on the individual, of which the work environment plays a major role. Acute, or short-term, stress causes an immediate reaction in the body. If the threat or demand passes quickly, the body generally returns to normal. However, with prolonged stress, many health problems can develop. Some of the early symptoms of stress-related problems include physical symptoms such as headaches, stomach problems, eating disorders, sleep disturbances, fatigue, muscle aches and pains, chronic mild illnesses; and psychological and behavioral symptoms such as anxiety, irritability, low morale, depression, alcohol and drug use, feeling powerless, isolation from co-workers. If exposure to stressors continues for a longer period of time, the individual may experience chronic health problems relating to physical conditions like high blood pressure, heart disease, stroke, spastic colon, impaired immune system, dysfunction, diabetes, asthma, musculoskeletal disorders; and psychological and behavioral problems like serious depression, suicidal behavior, domestic violence, alcohol abuse, substance abuse and burnout.

Nowadays, any individual’s work situation is highly demanding. Either he has to improve his career strength as and when required by the occupational demands or has to quit/maintain a low profile. The competitive era demands more from the individual employee than his actual ability. When the demand exceeds the capacity to fulfill it, the concerned person feels that the excessive demand is a burden, which is generally called occupational stress. The stress affects both the body and the mind either positively as motivation in its smallest amount or negatively as a burden in its highest amount of pressure that the individual cannot shoulder. In turn, that leads to physical and psychological problems. Occupational stress, hence, is found to be a mental and physical condition that calls in a detrimental effect on the individual’s productivity, effectiveness, personal health and quality of work (Comish and Swindle, 1994). Main components of this work-stress process are potential sources of stress (stressors), factors of individual differences (moderators/mediators) and consequences of stress (strain). Stressors (job-related and extra-organizational) are objective events; stress is the subjective aspect (Lu *et al*., 2003). Thus the concept of stress can best be understood by saying that some environmental variables (stressors) when interpreted by the individual (cognitive interpretation) may lead to stress (Dua, 1994, 59).

Occupational stressors have been researched widely and extensively during the past three or four decades. Hurrell *et al*. (1988) have identified some of the major stressors among executive personnel: (1) organizational practices (e.g., performance reward systems, supervisory practices, promotion opportunities), (2) job/task features (workload, work pace, autonomy), (3) organizational culture/climate (employee value, personal growth, integrity), (4) interpersonal relationships (supervisors, co-workers, customers) and (5) employee’s personal characteristics (personality traits, family relationships, coping skills). Burke (1988) and Lu *et al*. (2003) classified job stressors into 6 categories: Physical environment, role stressors, organizational structure and job characteristics, relationships with others, career development and work-family conflict. Copper *et al*. (1988) and Lu *et al*. (2003) are of the opinion that there are 6 sources of stress at work: Factors intrinsic to the job, management role, relationship with others, career and achievement, organizational structure and climate, and home/work interface. Other researchers (Antoniou *et al*., 2006) opine that stressors and either exogenous (i.e., unfavorable occupational conditions, excessive workload, lack of collaboration, etc.) or endogenous pressures (i.e., individual personality characteristics, etc.)[HREF1]. Studies on the impact of occupational stress found that acute and chronic stresses are major risk factors for the development and progression of coronary artery disease (Holmes *et al*., 2006), and job strain is found to be associated with coronary heart disease and hypertension risk in a number of occupations (Schnall *et al*., 1994). Elevations in systolic and diastolic blood pressures were found to be affected by the occupational stress due to exposure to psychosocial work (VanEgeren *et al*., 1992; Steptoe *et al*., 1999). In a study, Panagiotakos *et al*. (2003) proved the levels of occupational stress are positively associated with the risk of developing acute coronary syndromes, after systematically controlling for age, gender and region, and also high in the presence of, and the presence of smoking, hypertension, hypercholesterolemia, diabetes mellitus, physical activity status, educational and financial status and nutritional habits. Their study also observed that the presence of occupational stress seemed to affect more significantly males than females, smokers than nonsmokers, hypertensives than normo-tensives and high alcohol consumers compared to low alcohol consumers.

## Objectives

To find out whether there exist any differences between cardiac patients with coronary heart disease (CHD) and noncardiac patients with general illness with respect to mental health.

To find out whether there exist any differences between cardiac patients with coronary heart disease and noncardiac patients with general illness with respect to occupational stress.

## Hypothesis

Based on the objectives, the hypothesis that there will not be any significant difference between cardiac patients with CHD and noncardiac patients with respect to their occupational stress and mental health was formulated for statistical verification.

## MATERIALS AND METHODS

### Sample

The present study attempts to explore the status of mental health and occupational stress with respect to 2 categories of patients: Those who are suffering from cardiac problems and those who are suffering from noncardiac health problems. Occupational Stress Index (Srivastava, 1981) and Mental Health Questionnaire (WHO, 1984) were administered to both cardiac and noncardiac patients. The cardiac group consisted of 40 patients who were being treated at the cardiology department of a reputed hospital, and the noncardiac group consisted of 40 outpatients of the same hospital being treated for noncardiac problems like knee pain, headache, etc.

The patients of both groups were working as officers in state government departments located in Coimbatore city. Their ages ranged from 43 to 56 years, with more than 20 years of experience in the same department.

### Occupational stress index

The scale developed by Srivastava and Singh (1984) was used to measure the extent of job-related stress that patients of both categories perceived as arising from various constituents and conditions of their jobs. The items on the scale relate to most of the relevant components of a government official’s daily official work that can potentially cause stress. The authors explain that the instrument may be conveniently administered to employees of all levels working in various organizations. However, it is more suitable for employees working at the supervisory level and above. This scale has been found to have high reliability and has proved its validity through experiments; therefore, it was used for this study.

This index examines 12 particular dimensions:

1. Role overload: Role overload covers job situations like workload, staff insufficiency, lack of time, personal problems, job dissatisfaction, etc.
2. Role ambiguity: Role ambiguity is characterized by vague and insufficient information related to job role, vague and poor planning of job, vague expectations by colleagues and supervisors, etc.
3. Role conflict: Contradictory instructions from higher officers, interference of officials into the working conditions, vague instructions and insufficient facilities regarding new assignments, contradiction between office instructions and formal working procedures, difficulty in implementing new procedures and policies, etc., are included in this dimension.
4. Group and political pressures: This dimension covers the difficulty to adjust with the political and group pressures and formal rules and instructions, compulsion to perform unwillingly, maintenance of group conformity, violation of formal procedures and policies, etc.
5. Responsibility for persons: This dimension covers such aspects as the thrust of responsibility of other persons, the responsibility of other employees’ future, responsibility for the progress of organization, etc.
6. Under-participation: This dimension covers job areas such as the position of the person in the organization — that with high or low power; the acceptance of suggestions made by other persons, etc.
7. Powerlessness: This dimension covers areas such as acceptance of decisions taken by the person among employees, acceptance of suggestions regarding training programs of employees, lack of coordination of interest and opinion in making appointments for important posts, etc.
8. Poor peer relationships: The area covered under this dimension refers to poor interpersonal relationships with colleagues, colleagues’ attempt to defame and malign the employee as unsuccessful, colleagues’ lack of cooperation in solving administrative and industrial problems, lack of cooperation and team spirit of employees of the organization, etc.
9. Intrinsic impoverishment: Monotonous nature of assignments, opportunity to utilize abilities and experience independently, opportunity to develop aptitude and proficiency, place of suggestion in problem solving, etc., are included in this area.
10. Low status: This dimension covers respect received by an employee from others, the role of nature of the job in enhancing social status, due significance given by higher authorities to the post and work, etc.
11. Strenuous working conditions: This dimension covers tense circumstances in which work has to be done, risky and complicated assignments, unsatisfactory working conditions from the point of view of welfare and convenience, etc.
12. Unprofitability: Low salary, absence of rewards, lack of motivation, etc., are included here.

### Mental health questionnaire

This questionnaire was developed by the FilaBavi technical committee, which comprised of Swedish and Vietnamese epidemiologists, doctors and public health experts. This has been used to collect information about illness events and use of health services for executives. The questionnaire was tested among the respondents at different stages during a pilot study and then revised to be more appropriate to the local language and to common illness patterns. The English SRQ-20 was developed by the World Health Organization (WHO) as an instrument to screen for psychiatric disturbances. It consists of 20 questions which have to be answered by “Yes” or “No” depending on the presence or not of symptoms. Each question may score 0 or 1. It means that one can get a maximum score of 20. The SRQ-20 has been found to be reliable, valid and adaptable to screen for mental disorders in many countries, especially in the developing world (Araya *et al*., 1992; Harding, 1980; Mari and Williams, 1985, 1986b; WHO,1994).

## RESULTS AND DISCUSSION

Table 1 shows that noncardiac and cardiac patients significantly differed with regard to mental health (*t* value = *P* < 13.194). Cardiac patients (mean = 19.43, SD = 5.91) were found to have lower levels of mental health as compared to noncardiac patients (mean = 41.65, SD = 8.19).

**Table 1:** Mental health of noncardiac and cardiac patients

Group	*n*	Mean (SD)	Std. err. mean	‘*t*’ value
Noncardiac	40	41.65 (8.19)	1.35	13.194*
Cardiac	40	19.43 (5.91)	0.97	

* P < 05

Table 2 shows that cardiac patients were found to have higher levels of stress due to role ambiguity (mean = 15.22, SD = 4.685; *t* value = 4.028, *P* < 05), powerlessness (mean = 10.67, SD = 4.99; *t* value = 4.665,*P* < 05), intrinsic impoverishment (mean = 9.43, SD = 3.35;*t*value = 3.527,*P* < 05) and unprofitability (mean = 38.16, SD = 8.38; *t* value = 22.647,*P* < .05). Hence occupational stress of cardiac patients was found to be stemming from the vagueness of their job, too much of planning of the job, higher expectations by colleagues and supervisors, acceptance of poor decisions taken by other employees, acceptance of improper suggestions regarding training programs of employees, lack of coordination of interest and opinion in making appointments for important posts, monotonous nature of assignments, lack of opportunity to utilize abilities and experience independently, limited opportunity to develop aptitude and proficiency; and finally low salary, absence of rewards, lack of motivation also could be attributable for their stress.

**Table 2:** Occupational stress in noncardiac and cardiac patients

Occupational stress	Group	*n*	Mean (SD)	Std. err. mean	‘*t*’ value
Role overload	Noncardiac	40	1964 (590)	0.97	545*
	Cardiac	40	1362 (5.133)	0.84	
Role ambiguity	Noncardiac	40	11.38 (4.81)	0.79	4.02*
	Cardiac	40	15.22 (4.685)	0.77	
Role conflict	Noncardiac	40	1405 (4 77)	0.78	.12
	Cardiac	40	1391 (4 59)	0.75	
Unreasonable group and political pressures	Noncardiac	40	11.32 (4.64)	0.76	1.92
	Cardiac	40	9.70 (4.01)	0.65	
Responsibility for persons	Noncardiac	40	8.81 (3.96)	0.65	1.38
	Cardiac	40	10.24 (5 79)	0.95	
Under-participation	Noncardiac	40	10.24 (5 78)	0.95	2.17*
	Cardiac	40	7.81 (3.49)	0.57	
Powerlessness	Noncardiac	40	7.81 (3.49)	0.57	4.66*
	Cardiac	40	10.67 (4 99)	0.82	
Poor peer relations	Noncardiac	40	9.02 (3.83)	0.63	.72
	Cardiac	40	9 59 (338)	0.55	
Intrinsic impoverishment	Noncardiac	40	7.19 (2.62)	0.43	3.52*
	Cardiac	40	943 (335)	0.55	
Low status	Noncardiac	40	7.76 (3.46)	0.56	1.42
	Cardiac	40	9.13 (471)	0.77	
Strenuous working conditions	Noncardiac	40	6.97(2.33)	0.38	2.75*
	Cardiac	40	5.51(2.42)	0.39	
Unprofitability	Noncardiac	40	4 57 (2.21)	0.36	22.64*
	Cardiac	40	38.16 (8.38)	1.37	

* P < 05

The results also showed that noncardiac patients had higher levels of occupational stress due to role overload (mean = 19.64, SD = 5.90; *t* value = 5.45,*P* < 05), under-participation (mean = 10.24, SD = 5.78; *t* value = 2.17, *P* < 05) and strenuous working conditions (mean = 6.97, SD = 2.33; *t* value = 2.75, *P* <05). Noncardiac patients, who were suffering from noncardiac illnesses, were also found to be having stress due to job situations like excessive workload, staff insufficiency, lack of time, personal problems, job dissatisfaction, low-power position of the person in the organization, acceptance of improper suggestions made by other persons, tense circumstances in which work had to be done, risky and complicated assignments, unsatisfactory working conditions from the point of view of welfare and convenience.

The findings of the study imply that both cardiac and noncardiac patients have occupational stress, though from different types of sources. These areas can be improved upon at the work environment level itself so as to mitigate the levels of stress in the long run. Moreover, medical treatment can also focus on the psychosocial aspects of the sources of stress.

### Implications

Based on the findings, it is evident that associations between certain key dimensions of stressors and CHD were stronger among the executives. This is consistent with the findings that more robust work stress-CHD associations have been found in studies employing professional younger groups of executives as compared to those employing nonprofessional older cohorts (Theorell *et al*., 1998). Ensuring proper work environment, particularly providing very clear expectations of what a person is required to do at work, assigning adequate power to discharge duties, allocating tasks which utilize maximum internal strengths, enhancing perceived effort-profitability linkage at work will reduce the stress levels of employees sizably, and subsequently the occurrences of CHD can be prevented to the maximum extent. As these stressors are more critical and more likely to affect CHD, due care must be taken periodically by rendering remedial measures suitably, giving them the utmost priority.

Patients with CHD are more likely to have lower levels of mental health, perhaps due to the impact of stressors, as compared to noncardiac patients. Adopting appropriate changes in their work behavior and life style will lead to better mental health and enhanced quality of life.
